# *Schistosomiasis japonicum* in Indonesia: Progress and Surveillance Needs in Verge-of-Elimination Settings

**DOI:** 10.3390/tropicalmed11040086

**Published:** 2026-03-24

**Authors:** Achmad Naufal Azhari, Agrin Zauyani Putri, Ajib Diptyanusa, Sunardi Sunardi, Yayuk Agustin Hapsari, Regina Tiolina Sidjabat, Dauries Ariyanti, Zainal Khoirudin, Rezavitawanti Rezavitawanti, Herdiana Herdiana, Yullita Evarini Yuzwar, Farida Alhosani

**Affiliations:** 1 Global Institute for Disease Elimination (GLIDE), Abu Dhabi P.O. Box 764641, United Arab Emirates; 2 World Health Organization Indonesia Country Office, Jakarta 12940, Indonesia; agrinzauyani@gmail.com (A.Z.P.); diptyanusaa@who.int (A.D.); basrih@who.int (H.H.); 3 Directorate of Communicable Diseases, Ministry of Health of Republic of Indonesia, Jakarta 12950, Indonesia

**Keywords:** *Schistosomiasis japonicum*, near-elimination surveillance, Indonesia

## Abstract

*Schistosomiasis japonicum* transmission in Indonesia has declined substantially over recent decades, placing it in the last miles of elimination in the Western Pacific Region. As programmes transition from control to interruption of transmission, surveillance systems must be capable of detecting residual transmission. This study synthesised routine epidemiological data from 2015 to 2025 to assess Indonesia’s readiness for elimination and to identify key surveillance gaps in near-elimination settings. Descriptive quantitative analysis was conducted using national surveillance data from two endemic districts in Central Sulawesi, complemented by programme reports on mass drug administration, human diagnosis, animal reservoir surveillance, and snail surveys. Results showed that while prevalence in humans has remained low and responsive to mass drug administration, transmission persists through infected animal reservoirs and intermediate snail hosts. Surveillance performance is constrained by limited diagnostic capacity, inconsistent snail survey coverage, fragmented paper-based reporting systems, and weak integration across human, animal, and environmental sectors. These findings indicated that low prevalence in humans alone is insufficient to demonstrate interruption of transmission, particularly in zoonotic schistosomiasis. In conclusion, Indonesia’s experience highlights the need to strengthen near-elimination surveillance through sensitive diagnostics, integrated One Health approaches, and digitally enabled data systems to sustain elimination and support future verification of schistosomiasis transmission interruption.

## 1. Introduction

Neglected Tropical Diseases (NTDs) collectively affect more than one billion people worldwide, predominantly among the poorest and most marginalised populations [1]. Over the past decade, the global effort to eliminate NTDs has achieved significant progress, with a 32% reduction in people requiring interventions against NTDs compared to the 2010 baseline. This reflects significant gains in mass drug administration (MDA), morbidity control, and health system integration [2]. Schistosomiasis remains one of the world’s most persistent NTDs, affecting over 240 million people globally and placing more than 700 million at risk of infection in tropical and subtropical regions of Africa, Asia, the Caribbean, and South America. The disease is caused by parasitic trematodes of the genus Schistosoma. It continues to contribute substantially to morbidity, with chronic infection leading to anaemia, malnutrition, hepatosplenic disease, impaired development in children, and even death. The launch of the World Health Organization (WHO) Global NTD Roadmap 2021–2030 has reinvigorated global commitment to the elimination of schistosomiasis as a public health problem and eventual interruption of transmission. Achieving these goals requires sustained implementation of effective MDA with praziquantel, strengthened surveillance systems, vector control, case management, and integration with water, sanitation and hygiene (WASH) interventions, as well as veterinary public health [3,4].

**Figure 1:**
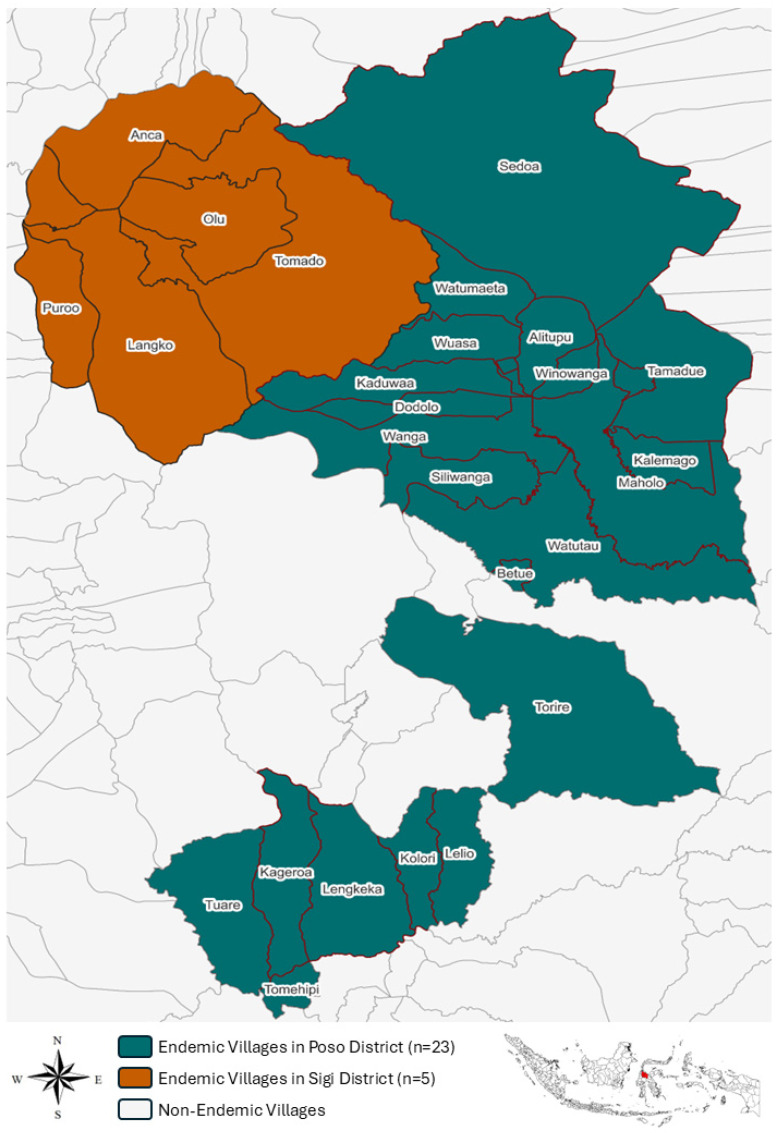
Geographic distribution of schistosomiasis-endemic villages in Central Sulawesi Province, Indonesia, 2024. The map illustrates 28 schistosomiasis-endemic villages identified through annual routine surveillance data, located in the Poso District (*n* = 23; dark teal) and Sigi District (*n* = 5; burnt orange). Villages shown in white represent non-endemic villages within the same geographical areas, where no indigenous transmission has been detected based on routine disease surveillance. The red area on the inset map indicates the geographic location of the study area within Indonesia. The figure was generated using QGIS version 3.40.15.

Within the WHO’s Western Pacific Region, two out of the six Schistosoma species are prevalent. *Schistosoma japonicum* remains endemic in China, the Philippines, and Indonesia. Meanwhile, *S. mekongi* is restricted to the Mekong River basin, affecting areas of Cambodia and the Lao People’s Democratic Republic (Lao PDR) [5]. The interruption of transmission in Thailand is yet to be formally confirmed [5]. Over the past two decades, the region has made substantial progress against schistosomiasis. In China, the transmission of *S. japonicum* has declined to historically low levels, with all endemic counties meeting the criteria for either elimination as a public health problem or interruption of transmission [6]. In the Philippines, infection remains focal and low in intensity, reflecting the impact of long-term MDA, environmental management, and health education programmes [7]. In Indonesia, the disease is distributed in only 28 villages across two districts in the Central Sulawesi Province, where prevalence has decreased to very low levels (<1%) following multisectoral interventions [8]. The spatial distribution of these endemic villages across the Central Sulawesi Province is presented in Figure 1, highlighting the highly focal nature of transmission and the geographic isolation of endemic foci within the province. Japan, where *S. japonicum* was previously endemic, has been consistently reported to be free of human and animal infections in the last few decades. Therefore, the country continues to serve as a model for successful elimination in the Pacific Region [9]. Collectively, these achievements suggest that the Asia Pacific region is approaching the final milestones of schistosomiasis elimination.

Among the three countries in the WHO Western Pacific Region endemic for *S. japonicum*, Indonesia presents a unique epidemiological profile characterised by highly focal and geographically isolated transmission areas. Satrija et al. reported that the disease was first identified in 1937, and a 1940 large-scale field survey found prevalence rates as high as 56% in three villages of the Lindu Valley in Central Sulawesi [10]. The intermediate snail host was later identified in 1971 and in 1973 named as *Oncomelania hupensis lindoensis*. Transmission studies in 1974 revealed the zoonotic nature of the disease, involving at least 13 mammalian hosts including rodents, wild deer, and wild pigs, as well as domestic animals such as cattle, water buffaloes, horses, and dogs. Indonesia’s schistosomiasis control efforts began in 1974 through selective treatment, snail control, and sanitation improvements. In 1982, the Ministry of Health launched a more coordinated national programme integrating MDA with praziquantel, environmental modification, and community mobilisation, leading to a decline in human infection from approximately 34% to below 1% by the late 1990s. Between 1999 and 2005, the integrated approach was further expanded under the Central Sulawesi Integrated Area Development and Conservation Project, achieving and sustaining infection levels below 1% in both human and snail populations [10]. Aligned with the National Medium-Term Development Plan (Rencana Pembangunan Jangka Menengah Nasional) 2025–2029, Indonesia aims to eliminate schistosomiasis in all 28 endemic villages by 2029 [11].

In support of this goal, routine schistosomiasis surveillance in Indonesia has consistently shown low prevalence of the disease, while also highlighting the need for more integrated surveillance and more sensitive diagnostic and stronger One Health frameworks to sustain the achievement. Building on these insights, this paper synthesises available epidemiological data and contextual programme information to identify gaps, opportunities, and strategic priorities for achieving and sustaining elimination in Indonesia and the Asia Pacific region. It further provides operational and policy recommendations relevant to other endemic countries transitioning from control to elimination of schistosomiasis japonica.

## 2. Materials and Methods

### 2.1. Study Design and Data Management

This paper employed a descriptive approach that was designed to assess Indonesia’s readiness for the validation of transmission interruption and identify operational and surveillance gaps. To synthesise the evidence, data were compiled from multiple sources—both published and unpublished—including epidemiological information from national and subnational surveillance records covering 2015–2025.

We covered the situation in two endemic districts for *S. japonicum* in the Central Sulawesi Province (Poso and Sigi Districts) representing different epidemiological and operational contexts to reflect variations in transmission intensity, health system capacity, and community participation. Out of 28 endemic villages, 17 villages in the Poso District represent areas with ecological and zoonotic complexity due to human–animal transmission cycles, while six villages in the same district represent a well-established control area with near-interruption of transmission but requiring sustained post-MDA surveillance. On the other hand, five endemic villages in the Sigi District represent remote, geographically challenging settings with limited access and community engagement barriers.

Routine surveillance and MDA data were collected from the Ministry of Health and annual country reports that are available in the WHO Global Health Observatory [12]. Collected data and information were analysed descriptively and thematically. Programme performance was compared against national elimination indicators (infection prevalence in humans and animals, treatment coverage, snail infection rate, and surveillance coverage). All findings were triangulated across data sources to ensure validity of the data. Microsoft Excel was used for data management, descriptive analysis, and the generation of tables and graphical outputs. The results of the analysis are presented in narrative form, supported by tables and illustrative pictures where appropriate.

### 2.2. Ethical Consideration

Data collection and analysis for this study relied solely on secondary data; hence, separate ethical clearance was not required. Official data-sharing approval was obtained from the Directorate of Communicable Diseases, Ministry of Health, Republic of Indonesia number, PM.03.03/C.III/4940/2025 dated 17 December 2025, and there are no personal identifiers or sensitive data presented in this publication.

## 3. Results

### 3.1. Surveillance in Humans, Mass Drug Administration, and Diagnostic Capacity

**Figure 2:**
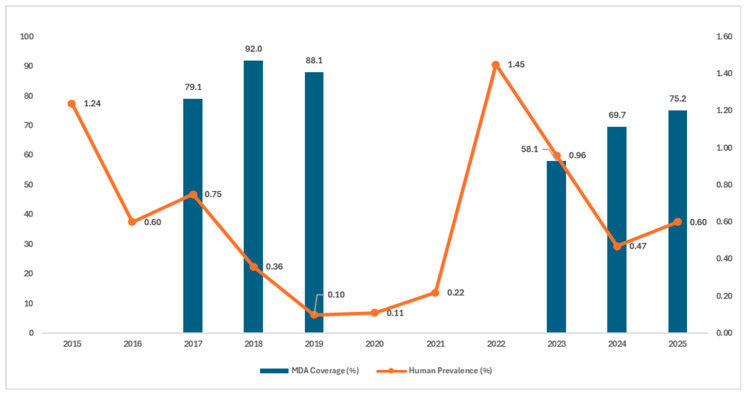
Human schistosomiasis prevalence and treatment coverage in Central Sulawesi Province, 2015–2025. Annual stool surveys were conducted in all 28 villages from 2015 to 2024. In 2025, six villages were excluded from the survey following five consecutive years of zero reported infections. Data sources: Ministry of Health, Republic of Indonesia and WHO PCT Databank.

The national schistosomiasis elimination programme in Indonesia is guided by Health Minister Decree Number 19 year 2018, which serves as an umbrella policy for the overarching schistosomiasis elimination efforts in the country. To operationalise the decree, the 2018–2025 National Roadmap for the Eradication of Snail Fever Disease (Schistosomiasis) was jointly launched by the Ministry of National Development Planning and the Ministry of Health in 2017. In accordance with the national roadmap, MDA with praziquantel was intensified during the 2017–2019 acceleration phase, targeting all individuals aged six years and above and achieving effective coverage in each year. All individuals residing in endemic villages were eligible for MDA, except pregnant women and those with severe illness. This led to a significant reduction in human prevalence, reaching 0.1% by the end of 2019. However, in 2020 and 2021, MDA was interrupted due to the Coronavirus disease-19 (COVID-19) pandemic and the “test and treat” approach was implemented, resulting in a gradual increase in schistosomiasis prevalence. By the end of 2022, the average prevalence across all endemic areas had risen to 1.45%, representing a 14.5-fold increase. In 2023, the national programme restarted MDA with suboptimal coverage; however, it still contributed to a reduction in schistosomiasis prevalence to 1%. In 2024, MDA was implemented again, covering almost 70% of the targeted population, resulting in a further decrease in prevalence to 0.47%. By the end of 2025, the national schistosomiasis elimination programme reported an MDA coverage of 75.2%, alongside a slight increase in prevalence to 0.6%. This increase should be interpreted in the context of surveillance design changes in 2025, when the number of specimens examined was substantially reduced following the exclusion of six villages that had reported zero cases for five consecutive years since 2021. With a reduced denominator and a relatively stable number of positive cases, the calculated prevalence increased, reflecting a methodological effect rather than a true recrudescence in Central Sulawesi and nationally. The temporal relationship between human prevalence and MDA coverage from 2015 to 2025 is illustrated in Figure 2, demonstrating the close association between programme performance and fluctuations in infection levels.

Annual cross-sectional prevalence surveys were conducted in all 28 endemic villages using the Kato–Katz thick-smear method, targeting all registered residents aged 2 years and above. The Kato–Katz method was applied in accordance with WHO recommendations for the detection of schistosomiasis infection and quantification of infection intensity. Survey performance was strongly influenced by community participation in stool sample collection and varied substantially by village and year, with participation ranging from 14,568 to 21,303 individuals per year, as summarised in Table 1. Several factors were identified as influencing the community participation, including community fatigue due to repeated multi-year and multi-day stool collection activities, the COVID-19 pandemic, community perceptions of a substantial decline in schistosomiasis prevalence, and challenges in accessing households in very remote areas.

**Figure 3:**
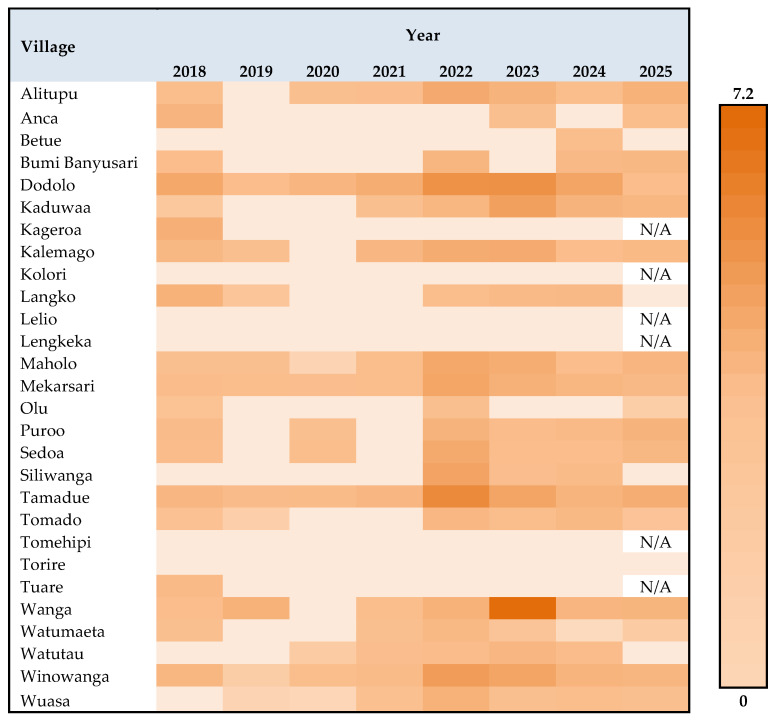
Village-level variation in schistosomiasis prevalence (%) by year, 2018–2025. The heatmap illustrates annual prevalence across endemic villages during the period 2018–2025, with colour intensity indicating increasing prevalence. This figure highlights both temporal trends and persistent heterogeneity in transmission across villages.

Considerable heterogeneity in prevalence across endemic villages was observed over time, despite relatively district-level prevalence. While overall prevalence remained below 2% in most years, village-level data showed fluctuating transmission patterns and persistent clustering in specific villages. As illustrated in Figure 3, the heatmap visualises annual prevalence across all 28 endemic villages from 2018 to 2025 and demonstrates that several villages experienced noticeable increases in prevalence during 2021–2022 following the interruption of MDA, whereas others consistently reported zero or near-zero transmission throughout the observation period. In 2025, six villages are indicated as “N/A” due to their exclusion from routine surveys after five consecutive years of zero reported infection.

**Figure 4:**
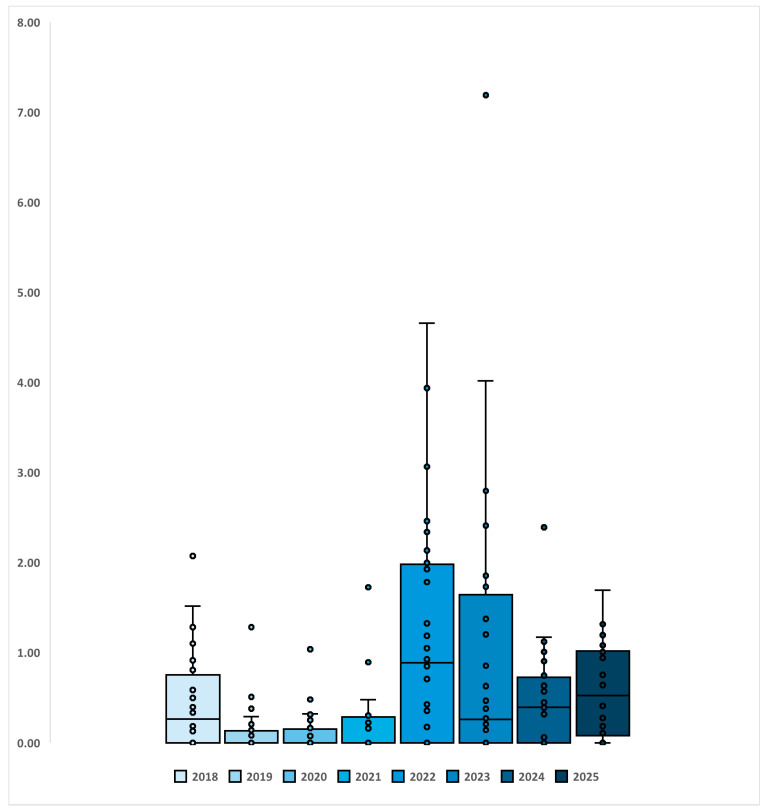
Between-village distribution of schistosomiasis prevalence by year, 2018–2025. Box-and-whisker plots summarise village-level prevalence for each survey year. Boxes represent the interquartile range (IQR), the horizontal line within each box indicates the median, whiskers denote the spread of values across villages (up to 1.5 × IQR), and individual points represent village-specific prevalence estimates. This figure highlights temporal changes in both median prevalence and heterogeneity between villages across survey years.

To further illustrate temporal changes in central tendency and dispersion across villages, Figure 4 presents box-and-whisker plots summarising annual village-level prevalence distribution from 2018 to 2025. The figure shows a general decline in median prevalence following intensified MDA, particularly after intensified MDA in 2018 and a rebound in 2022, while also revealing substantial variability between villages within each survey year. Although the interquartile range narrowed in several post-MDA years, the persistence of outliers indicates that certain villages continued to report higher prevalence than others.

The major surveillance gaps included the absence of infection intensity data, limited capacity-building for laboratory personnel, and heavy workloads that constrained the routine quantification of infection intensity. Heavy-intensity infections were not routinely measured from 2015 to 2022, as the current diagnostic capacity remains limited to basic microscopy that distinguishes positive and negative results only. This prevents alignment between national indicators and WHO Roadmap requirements. This gap began to be addressed in 2023, when the programme started collecting moderate- and heavy-intensity infection data, with only 2 of the 81 positive cases (2.47%) identified as high intensity by the end of 2024. Additionally, all data—from human surveys to snail and animal surveillance—remain fragmented and paper-based, with limited digitisation and no central repository for integrated analysis. While computers are available in schistosomiasis laboratories, electrical power interruptions, limited feedback loops, and provincial-level data processing mean that results rarely return to local laboratories for interpretation and action.

Additional diagnostic gaps include the absence of formal quality assurance and quality control mechanisms and the limited availability of sensitive screening tools beyond stool microscopy. In 2023, the WHO introduced recombinant-antigen ELISA (RA-ELISA) to improve screening sensitivity in near-elimination settings, where the performance of Kato–Katz is reduced at low prevalence. However, RA-ELISA is still undergoing evaluation by other institutions in endemic areas of Indonesia, and nationwide laboratory quality assurance systems, including external proficiency testing for ELISA and molecular diagnostics, are not yet fully institutionalised. As prevalence declines further, the lack of regional capacity for real-time polymerase chain reaction (qPCR) also limits confirmatory testing of microscopy-positive results.

### 3.2. Reservoir Host Surveillance

Animal reservoir surveillance for *S. japonicum* in Indonesia is implemented as part of routine zoonotic disease surveillance but remains irregular, under-resourced, and inconsistent across endemic areas. Infection in animals is diagnosed primarily through parasitological stool examination conducted by the Maros Veterinary Research Centre under the Ministry of Agriculture. Surveillance activities focus mainly on domesticated animals, including cattle, buffaloes, pigs, horses, and dogs. They are conducted intermittently, depending on resource availability and operational feasibility. Surveys use a purposive sampling approach, targeting domesticated animals owned by consenting households, and therefore cover only a proportion of the total animal population. Animals found to be positive are treated using combination anthelmintic formulations containing praziquantel and albendazole. However, systematic or preventive praziquantel treatment of livestock has not been implemented at scale, largely due to logistical constraints and the absence of praziquantel formulations registered for cattle in Indonesia.

In addition to domestic animals, at least 13 mammalian species—including rodents, deer, and civet cats—are recognised as susceptible zoonotic hosts, although financial constraints have limited comprehensive surveillance of these reservoirs. Available data from the Maros Veterinary Research Centre indicate that infection has been most consistently detected in cattle, as presented in Table 2.

In 2023, an additional animal survey was conducted by the Ministry of Health, focusing on rodents as a proxy indicator for the broader reservoir host situation. This survey was integrated with a snail survey and showed a significant increase in infection rate among rodents across the Poso and Sigi Districts with an overall infection rate of 31.6%, as summarised in Table 3. The findings from this survey varied substantially across villages and ecological zones with the infection rate ranging from 0% to 100%. This indicates that zoonotic transmission remains active and highly heterogeneous across villages. It also underscores the need for strengthened surveillance, expanded sampling coverage, and integration with broader zoonotic host monitoring in order to better characterise the animal reservoir burden in Central Sulawesi.

Although the schistosomiasis elimination programme in Indonesia is formally designed as a multisectoral One Health initiative, involving the health, agriculture, public works, environment, and village development sectors as mandated under Health Minister Decree Number 19 year 2018, operational coordination across sectors remains uneven. In practice, human MDA and snail control activities are more consistently implemented than veterinary interventions targeting animal reservoirs.

At present, standardised praziquantel formulations and dosing regimens for domestic animals are not available in Indonesia, and systematic treatment of livestock has therefore not been implemented. While treatment of infected animals is recognised as a critical component for interrupting zoonotic transmission, veterinary interventions remain largely ad hoc and constrained by regulatory and logistical barriers. This gap represents a major limitation in Indonesia’s efforts to interrupt *S. japonicum* transmission and highlights the need to prioritise the development and operationalisation of feasible strategies for treating domestic animals in endemic settings.

### 3.3. Vector Surveillance

*Oncomelania hupensis lindoensis* is the specific subspecies of freshwater snail that plays the intermediate host role in schistosomiasis transmission in Indonesia. The natural breeding place of this snail is a shallow waterlogged area surrounded by vegetation that prevents direct sunlight exposure such as untended coffee plantations and rice fields, springs, waterways, and the buffer zone of Lore Lindu National Park. The snails release a large number of cercariae, the infectious form of the parasite, into water bodies that can penetrate human and other mammals’ skin. Snail survey and mapping have been implemented multiple times in the last decade, including most recently in 2023. This activity is crucial in the schistosomiasis elimination programme to estimate the level of active disease transmission within an area, even in a low-prevalence setting. However, the performance of snail surveillance activities is highly dependent on the availability of funds, resulting in inconsistent selection of targeted villages and foci areas, as illustrated in Table 4.

In addition to the number of snails and their infection rate, the number of foci or snail habitats distributed across two endemic districts is another key indicator monitored by the national schistosomiasis programme. During the acceleration phase of the roadmap (2017–2019), several snail-control activities were implemented, including environmental modifications, such as the revitalisation or construction of irrigation channels and chemical control using molluscicide. According to routine programme monitoring data from the National NTD Programme, these efforts successfully reduced the number of snail foci from 301 in 2017 to 224 in 2021, equivalent to a 25.6% reduction.

## 4. Discussion

### 4.1. Operational Implication for Human Surveillance, Mass Drug Administration, and Diagnostic Capacity

In Indonesia, human surveillance of schistosomiasis is characterised by fluctuating prevalence trends over decades, strongly influenced by MDA performance. Available programme surveillance data suggest that the disruption of annual MDA, for example due to COVID-19 pandemic, likely contributed to a temporary increase in infection risk, particularly in villages with persistent environmental and limited vector control activities. Regional evidence from China and the Philippines confirm that, if control efforts are weakened transmission of *S. japonicum* will rebound to previous levels. In China, the long-term analysis showed prevalence declined, slowed down or reversed during periods of reduced preventive chemotherapy and environmental interventions, with several documented rebounds following gaps in the large-scale treatment [14]. Serological and field studies similarly capture the fluctuation in the infection of schistosomiasis in China that is associated with inconsistent annual control activities. This underscores the need for sustained and uninterrupted programmes, particularly preventive chemotherapy [15]. These transmission dynamics also align with the local observation from endemic lake and marshland areas in the Philippines, where persistent *S. japonicum* transmission and a lower MDA coverage are observed [7].

This pattern is also consistent with global evidence across all major Schistosoma species, not only *S. japonicum*. In Indonesia, the transition from MDA to selective treatment during 2020–2021 was followed by signs of increased infection risks in some villages. These trends were particularly evident in settings with sustained environmental and animal transmission and limited control of snail foci. This aligns with modelling work from African settings showing that postponement of even a single MDA round can lead to higher Schistosoma prevalence and delay the achievement of its elimination as a public health problem, especially in moderate-to-high transmission areas [16]. Empirical data from Zanzibar further demonstrate recrudescence of *S. haematobium* infections after a one-year treatment gap following multiple rounds of MDA, with prevalence in school-aged children rising substantially after the interruption [17]. In Brazil, programme data from endemic states in the Northeast documented sharp reductions in the population surveyed, tests performed, and treatment delivered in 2020–2021, reflecting service disruption and reduced access to diagnosis and care, a pattern that mirrored Indonesia’s experience during the pandemic [18]. Against this backdrop, the resumption of MDA in Indonesia in 2023–2024 mirrors experiences from other preventive chemotherapy NTD programmes, where countries such as Guinea and those described by Itaye et al. were able to restart MDA with adapted delivery strategies while attempting to contain the indirect impact COVID-19 had on transmission [19]. MDA recovery is essential to regain lost ground towards the WHO 2030 targets.

In the area of diagnosis, all major reviews indicate that the sensitivity of microscopy drops sharply as transmission decreases for all major schistosome species [20,21,22,23]. Indonesia reported extremely low prevalence in humans (<1%) in the last decade, particularly when intensified MDA is implemented with high coverage. However, this prevalence relies solely on Kato–Katz, without more sensitive tests for screening and confirmation, which may suggest the risk of underdiagnosis. This is particularly concerning where community participation in stool surveys remains inconsistent and in areas where transmission persists in reservoir hosts. To address the diagnostic gap, the Indonesia Research Partnership on Infectious Diseases (INA-RESPOND) validated the Point-of-Care Circulating Cathodic Antigen (POC-CCA) as an alternative diagnostic method. Although the final result of this study has not yet been published, the interim reports indicate that the reliability of POC-CCA with urine samples for *S. japonicum* is questionable with a high number of false positives (low specificity) and further investigation is required [24].

Over a decade ago, recombinant-antigen Enzyme-Linked Immunosorbent Assay (RA-ELISA) for *S. japonicum* demonstrated substantially higher sensitivity than stool microscopy, particularly for low-intensity infections. Early studies in the Philippines showed that thioredoxin peroxidase-1 (SjTPx-1) and tandem-repeat antigens, notably Sj7TR, achieved high sensitivity and specificity in human sera, outperforming crude-antigen ELISAs and detecting infections missed by Kato–Katz microscopy [25]. Subsequent evaluations confirmed their robust diagnostic performance across multiple cohorts [26] and in reservoir hosts, where they performed comparably or better than PCR [27]. Together, these findings underscore the importance of incorporating recombinant-antigen ELISAs as more sensitive tools for surveillance in low-transmission settings. The introduction of the RA-ELISA in 2023 in Indonesia presents a new opportunity not only to enhance schistosomiasis screening and mapping but also to strengthen Indonesia’s broader health system through a more integrated surveillance approach [28].

While more sensitive diagnostics are essential for surveillance in near-elimination settings, evidence from multiple endemic countries shows that the reliability of schistosomiasis elimination surveillance systems also critically depends on technician expertise and robust QA and QC mechanisms [21,29,30]. These factors and patterns closely mirror the situation in Indonesia, where the lack of QA/QC mechanisms, irregular microscopy training, and the absence of competency certification for schistosomiasis laboratory technicians are observed. In progressing toward the interruption of schistosomiasis transmission from its status of elimination as a public health problem, Indonesia’s diagnostic constraints highlight the need for tools that are not only more sensitive but also consistently reliable. The experiences from other countries with similar epidemiological characteristics such as China and Philippines revealed that molecular methods, particularly qPCR, have consistently demonstrated superior performance in the detection of low-intensity schistosome infection [31,32]. The test is able to reveal substantial levels of residual transmission that were not captured by traditional microscopy [31,32]. This regional evidence indicates that integrating qPCR into Indonesia’s surveillance framework would substantially strengthen case detection, provide a more reliable assessment of ongoing transmission, and support verification of elimination targets.

The final observation on human surveillance systems for the schistosomiasis elimination programme in Indonesia is the heavy reliance on paper-based recording and reporting mechanisms with limited linkage to national electronic health information systems. In routine practice, this situation means that schistosomiasis data are compiled separately from other communicable diseases, aggregated manually, and often disseminated with limited analysis and not in a timely manner. Similar trends and weaknesses have been documented in other countries and regions, where fragmented recording and reporting tools, incomplete case registers, and weak feedback mechanisms undermine the performance of overall post-MDA surveillance, particularly at subnational levels [33]. The transition from paper-based, standalone systems to more integrated electronic systems, such as District Health Information System-2 (DHIS-2)-based reporting or internet-linked reporting modules, has improved the completeness and timeliness of schistosomiasis surveillance in endemic countries including Kenya and China, provided it is supported by clear guidelines, adequate capacity-building and sustained resources for system maintenance [34,35,36]. Furthermore, the global normative guidance now explicitly calls for more integrated monitoring and evaluation systems for NTDs, rather than traditional parallel reporting channels [37]. Based on these backgrounds, the current schistosomiasis surveillance system in Indonesia is not only labour-intensive but also structurally constrained in its ability to generate timely, high-quality data, reinforcing the need to integrate schistosomiasis indicators into the routine electronic communicable disease platform and to invest in data management capacity at all levels.

### 4.2. Strengthening Reservoir Host Surveillance Within a One Health Framework

Although routine animal surveillance is not captured within the Ministry of Health’s public surveillance system, publicly available datasets from the Ministry of Agriculture provide the only continuous cross-species surveillance record for zoonotic *S. japonicum* transmission in Indonesia, documenting systematic faecal examinations of livestock and companion animals in endemic areas between 2018 and 2023. Analysis shows persistent animal infection in the Poso and Sigi Districts, with annual fluctuations but no complete interruption of transmission, predominantly in buffaloes and cattle, followed by pigs, horses and dogs. This distribution reflects both species-specific exposure patterns and the greater ease of collecting faeces from cattle [13,38]. These findings are consistent with earlier studies in Indonesia demonstrating the sustained role of large ruminants and domestic animals as key reservoirs for *S. japonicum* transmission [39]. Taken together, this evidence illustrates a stable but low-level persistence of zoonotic infections despite intensive MDA efforts in humans, underscoring the importance of integrating animal surveillance into national schistosomiasis monitoring frameworks and recognising livestock as continuous contributors to environmental contamination in these foci.

Several historical studies from schistosomiasis-endemic areas in Indonesia further reinforce the longstanding role of domestic animals as key reservoirs sustaining *S. japonicum* transmission in Central Sulawesi. Detailed field investigations in the Sigi District documented natural infection in buffaloes, cattle, horses, pigs and dogs, with buffaloes showing the highest prevalence and contributing the greatest daily egg output into the environment [40]. The study also showed that grazing patterns of large ruminants frequently brought them into contact with known snail habitats, particularly irrigated rice fields and marshlands, creating continuous opportunities for both infection and environmental contamination. Notably, animal infections persisted despite ongoing human treatment activities, indicating that transmission is maintained through a multi-host zoonotic system rather than human infection alone. These findings, together with earlier evidence of infection in wild rodents [41] and more recent confirmation of domestic reservoir contributions [42], highlight that Indonesia’s schistosomiasis foci have always been characterised by a resilient zoonotic reservoir that requires integrated animal–human surveillance for the effective interruption of transmission. Targeted animal-based MDA, particularly for bovines, can substantially reduce environmental contamination and transmission pressure, supporting its consideration as a complementary strategy to human MDA in zoonotic foci such as those in Indonesia [42,43].

Interrupting the transmission of *S. japonicum* requires a strategy shift from single-sector interventions to integrated and coordinated approaches under the One Health framework that explicitly links human, animal, and environmental health [44]. This need reflects the parasite’s complex zoonotic lifecycle involving humans, multiple domestic mammals and *Oncomelania* snails, with evidence consistently showing that large ruminants contribute disproportionately to environmental egg contamination, as observed in endemic settings in China and the Philippines, where cattle and water buffaloes act as dominant amplifying hosts [45,46]. In Indonesia, free-grazing livestock practices, unmanaged animal movement and agricultural water use facilitate frequent contact between uncaged animals and *Oncomelania hupensis lindoensis* snail foci, sustaining transmission at the human–animal–environment interface. Evidence from endemic areas indicates that improved livestock management, including controlled grazing, strategic caging and integrated veterinary interventions, can significantly reduce animal–snail contact and environmental contamination, supporting the operational value of One Health-based control strategies [39,47,48]. Strengthening One Health integration in Indonesia therefore requires routine animal surveillance alongside human surveys, enhanced diagnostic capacity for livestock, and institutionalised inter-ministerial data sharing between the Ministry of Health, the Ministry of Agriculture and environmental agencies.

### 4.3. Strengthening Vector Surveillance in Near-Elimination Settings

Despite the considerable reductions in reported human schistosomiasis prevalence, persistent infection is consistently observed through animal reservoir and snail surveillance. This indicates the transmission risk remains unchanged in schistosomiasis-endemic areas in Indonesia. Evidence from other endemic countries with similar zoonotic settings shows that low prevalence in humans does not necessarily equate to the interruption of transmission, particularly where animal reservoirs and environmental contamination persist [5,49]. In such a context, residual transmission can remain undetected by routine human surveillance alone and can lead to rapid recrudescence if control efforts are relaxed [50].

Data and information on snail surveillance provided in this paper highlight that the inconsistency of snail surveillance coverage is considered as one of critical challenges in the schistosomiasis elimination programme in Indonesia. The annual snail surveys have varied substantially over time and across locations, highly influenced by budget availability, competing programme priorities, and operational challenges in remote areas. These further raise concerns that some active or emerging transmission foci may be missed [51,52]. Furthermore, evidence from different elimination settings highlight that incomplete or irregular snail surveillance can lead to an underestimation of transmission intensity and delayed detection of recrudescence, particularly when surveillance is not systematically linked to historical foci and environmental risk mapping [50,53].

Snail control remains a cornerstone intervention for the interruption of schistosomiasis transmission and must be implemented strategically alongside routine surveillance. Chemical approaches with molluscicides, when applied in a targeted and evidence-based manner, have proven to significantly reduce snail populations [54,55]. However, heavy reliance on molluscicides alone is insufficient as snail populations may rapidly recolonise if environmental determinants are not addressed. Additionally, it provides another risk to the environment, namely chemical pollution and potential insecticide resistance. Integrating snail control within broader environmental management and land-use planning such as drainage improvement, vegetation management, and water flow regulation are therefore essential for long-term impact [55].

Sustained and systematic snail mapping is a critical enabling component for both surveillance and control efforts. Updating spatial maps of snail habitats on a routine basis allows programmes to identify persistent and newly emerging foci, prioritise interventions, and optimise allocation of limited resources [53,56]. For Indonesia’s context, sustaining and strengthening snail surveillance with ecological data, risk, and indicators and linking them to overall schistosomiasis surveillance platforms will be crucial to close the remaining transmission gaps and generate the evidence required to demonstrate the interruption of transmission.

### 4.4. Next Steps and Required Surveillance System for Interruption of Transmission

Routine programme surveillance data from endemic districts in the Central Sulawesi Province indicates that prevalence in humans has consistently been recorded below 2%. This reflects the cumulative impact of annual preventive chemotherapy, snail control, health education, and targeted environmental management implemented since the 1980s. This achievement highlights a marked improvement compared to historical prevalence levels, which once exceeded 10% in several endemic villages, and currently places Indonesia among countries approaching the elimination threshold for schistosomiasis [10,57].

As the country advances from low prevalence towards the interruption of transmission, the surveillance systems must evolve to address the inherent limitation of traditional control-oriented approaches in near-elimination settings. As discussed earlier, standard parasitological methods, including Kato-Katz thick-smear microscopy, which have insufficient sensitivity to reliably detect low-intensity and sporadic infections, will increase the risk of undetected residual transmission. Studies and evidence from elimination-focused surveillance approaches indicate that programmes entering the “end game” require a paradigm shift toward surveillance systems explicitly designed to demonstrate the absence of transmission rather than a reduction in disease burden. This transition requires the incorporation of more sensitive diagnostic tools, such as serological and molecular assays, to complement routine microscopy and enhance early detection of ongoing transmission foci [51,58]. Additionally, the optimisation of AI-based diagnosis machines [59] and the innovative “one prick, many tests” approach, which has successfully demonstrated how multi-disease surveillance could reduce costs, strengthen efficiency, and increase community participation, could be considered by the national programme, aiming for the same objective [60,61].

In addition to technological innovation, strengthening surveillance in near-elimination settings requires an operational approach that enhances coverage, sensitivity, and sustainability at the community level. Evidence from a community-based study in Central Sulawesi revealed that community-embedded surveillance mechanisms can function as an effective extension of formal surveillance systems in low-prevalence contexts. The implementation of the village-based “Peda Team” model, which trained local community cadres to support stool sample collection, conduct snail and rodent surveillance, assist with and improve the quality of MDA implementation, and report findings on a routine basis, was associated with substantial improvements in overall surveillance performance, including stool examination coverage exceeding 80% in most endemic villages and a significant reduction in identified snail focus areas. Importantly, this initiative was institutionalised through village regulations, enabling surveillance functions to be maintained beyond the national allocated budget and externally funded projects. This experience highlights that community engagement, when systematically embedded into established surveillance systems, is able to enhance the sensitivity and sustainability of detection efforts in the last-mile phase [62].

Given the zoonotic and environmentally mediated transmission of *S. japonicum*, the interruption of transmission cannot be achieved through surveillance in humans alone. Evidence from multiple endemic settings demonstrate that animal reservoirs and infected snail populations may persist even when prevalence in humans approaches zero. They serve as silent sources of recrudescence [5,17]. As such, future surveillance systems must systematically integrate human, animal, and environmental surveillance components within a One Health framework.

In Indonesia, schistosomiasis transmission is likely sustained primarily through animal reservoirs and infected snail habitats, with humans acting largely as incidental hosts; consequently, interventions focused solely on human mass drug administration are unlikely to be sufficient to interrupt transmission. Future efforts should therefore prioritise strategies that address the zoonotic and environmental determinants of transmission. In line with the National Roadmap on Schistosomiasis Eradication, these include integrated snail control through environmental modification and targeted molluscicide; strengthened surveillance and management of animal reservoirs; improvements in water, sanitation, and hygiene (WASH) infrastructure; and community-based environmental and livestock management, supported by strengthened coordination across health, agriculture, environment, and village development sectors.

From a system and policy perspective, interrupting transmission requires a more advanced surveillance architecture that is standardised, risk-based and digitally integrated to enable cross-sector interoperability and timely analysis, thereby generating actionable outputs aligned with emerging global expectations for elimination verification [2,18]. However, the lack of harmonised global tools to assess progress towards elimination as a public health problem and interruption of transmission, particularly for zoonotic schistosomiasis, remains a recognised gap, as highlighted in the WHO Global NTD Report 2025, which notes that validation and verification frameworks are still under development [2]. Aligning national surveillance systems with these global standards will be critical for Indonesia and similar Western Pacific settings to demonstrate elimination, sustain gains and reduce the risk of recrudescence.

### 4.5. Study Limitations

This study has several limitations. Analyses were based on routine surveillance data reported in aggregated form, which precluded age- and occupation-specific analyses and limited assessment of individual-level risk factors. Village-level survey coverage varied by year, with incomplete data for some villages in 2015, 2016, 2017, and 2025, which may affect comparability across years. In addition, reliance on Kato–Katz diagnostics, while programmatically appropriate, may underestimate low-intensity infections in near-elimination settings. Nevertheless, the large annual sample sizes and the consistent surveillance approach applied over time provide a robust overview of temporal trends and spatial heterogeneity of human schistosomiasis in Indonesia.

## 5. Conclusions

Indonesia has made substantial progress and contributed to the Western Pacific region’s movement towards the elimination of *S. japonicum*. However, synthesis of human, snail and animal surveillance data indicates that residual zoonotic transmission persists, threatening the sustainability of elimination gains. Disruptions to mass drug administration during the COVID-19 pandemic and subsequent rebounds in human prevalence underscore the fragility of progress when surveillance and control efforts weaken. Key gaps include limited diagnostic sensitivity, inconsistent snail surveillance, fragmented paper-based reporting and suboptimal integration across human, animal and environmental sectors, constraining the programme’s ability to reliably demonstrate elimination.

As Indonesia transitions from control to the final stages of elimination, surveillance systems must shift from measuring disease burden to detecting residual transmission and preventing recrudescence. Strengthening sensitive diagnostics, implementing integrated One Health surveillance and adopting digitally enabled data systems are critical strategic priorities, alongside piloting innovative tools such as serological, molecular, and AI-assisted approaches. Repositioning schistosomiasis elimination as an inter-sectoral public good, rather than a vertical programme, and aligning national surveillance with WHO validation and verification frameworks will be essential to sustain political and financial commitment. Indonesia’s experience demonstrates both the feasibility and the challenges of interrupting transmission of schistosomiasis, offering transferable operational and policy lessons for other endemic countries in the Western Pacific region approaching elimination.

## Figures and Tables

**Table 1:** Annual human schistosomiasis survey results, 2018–2025.

Year	Number Examined (*n*)	Number Positive (*n*)	Prevalence (%)	95% CI
2018	19,384	70	0.36	0.29–0.46
2019	20,117	20	0.10	0.06–0.15
2020	21,303	24	0.11	0.08–0.17
2021	21,084	47	0.22	0.17–0.30
2022	17,664	256	1.45	1.28–1.64
2023	17,215	166	0.96	0.83–1.12
2024	17,120	81	0.47	0.38–0.59
2025	14,421	87	0.60	0.49–0.74

**Table 2:** Results of animal surveys in Central Sulawesi Province, 2020 (Data source: Maros Veterinary Research Centre [13]).

District	Animal	Prevalence by Year (%)					
		2018	2019	2020	2021	2022	2023
Poso	Buffalo	0.00	0.95	1.03	0.00	0.00	0.00
	Cow	0.00	0.08	0.00	0.00	15.49	5.43
	Pig	0.00	0.00	5.24	0.00	0.00	0.00
	Dog	0.00	0.00	1.11	0.00	0.00	0.00
	Total	0.00	0.12	1.37	0.00	6.49	2.72
Sigi	Buffalo	0.00	2.63	0.00	0.00	0.00	0.00
	Cow	5.88	0.00	0.00	0.00	0.52	0.00
	Pig	0.00	0.00	0.00	0.00	0.00	0.00
	Dog	0.00	0.00	0.00	0.00	0.00	0.00
	Horse	N/A	1.39	0.00	0.00	0.00	0.00
	Total	2.56	0.79	0.00	0.00	0.17	0.00

**Table 3:** Result of rodent surveys in Central Sulawesi Province, 2023 (Data source: Ministry of Health, Republic of Indonesia).

District	Number of Samples Collected	Number of Positives	Prevalence (%)
Poso	36	22	61.1
Sigi	40	2	5.0
Total	76	24	31.6

**Table 4:** Result of snail surveys in two endemic districts, 2017–2023 (Data source: Ministry of Health, Republic of Indonesia).

Year	Poso District		Sigi District	
	Number of Snails Examined	Infection Rate (%)	Number of Snails Examined	Infection Rate (%)
2017	609	6.24	699	3.43
2021	4151	5.23	143	0.70
2023	2441	5.49	127	11.02

## Data Availability

No new data were generated for this study and data sharing is therefore not directly applicable. The schistosomiasis surveillance data supporting the findings of this study are not publicly available but may be obtained from the Directorate of Communicable Diseases, Ministry of Health, Republic of Indonesia, upon reasonable request and subject to approval.
